# Recombinant HIV-1 vaccine candidates based on replication-defective flavivirus vector

**DOI:** 10.1038/s41598-019-56550-4

**Published:** 2019-12-27

**Authors:** M. Giel-Moloney, M. Esteban, B. H. Oakes, M. Vaine, B. Asbach, R. Wagner, G. J. Mize, A. G. Spies, J. McElrath, M. Perreau, T. Roger, A. Ives, T. Calandra, D. Weiss, B. Perdiguero, K. V. Kibler, B. Jacobs, S. Ding, G. D. Tomaras, D. C. Montefiori, G. Ferrari, N. L. Yates, M. Roederer, S. F. Kao, K. E. Foulds, B. T. Mayer, C. Bennett, R. Gottardo, M. Parrington, J. Tartaglia, S. Phogat, G. Pantaleo, H. Kleanthous, K. V. Pugachev

**Affiliations:** 1 Sanofi Pasteur, Cambridge, MA 02139 USA; 2 Centro Nacional de Biotecnología (CNB-CSIC), Madrid, Spain; 3 University of Regensburg (UREG), Institute of Medical Microbiology and Hygiene, 93053 Regensburg, Germany; 4 Fred Hutchinson Cancer Research Center (FHCRC), Seattle, WA 98109 USA; 5 Service of Immunology and Allergy, Department of Medicine, Lausanne University Hospital, 1011 Lausanne, Switzerland; 6 Infectious Diseases Service, Department of Medicine, Lausanne University Hospital, 1011 Lausanne, Switzerland; 7 Bioqual Inc, Rockville, Maryland 20850 USA; 8 Arizona State University (ASU), Tucson, AZ 85745 USA; 9 EuroVacc, Amsterdam, The Netherlands; 10 Duke University Medical Center, Durham, North Carolina 27710 USA; 11 Vaccine Research Center, NIAID, NIH, Bethesda, MD 20892 USA; 12 University Hospital Regensburg, Institute of Clinical Microbiology and Hygiene, 93053 Regensburg, Germany

**Keywords:** Molecular engineering, Preclinical research, Molecular medicine

## Abstract

Multiple approaches utilizing viral and DNA vectors have shown promise in the development of an effective vaccine against HIV. In this study, an alternative replication-defective flavivirus vector, RepliVax (RV), was evaluated for the delivery of HIV-1 immunogens. Recombinant RV-HIV viruses were engineered to stably express clade C virus Gag and Env (gp120TM) proteins and propagated in Vero helper cells. RV-based vectors enabled efficient expression and correct maturation of Gag and gp120TM proteins, were apathogenic in a sensitive suckling mouse neurovirulence test, and were similar in immunogenicity to recombinant poxvirus NYVAC-HIV vectors in homologous or heterologous prime-boost combinations in mice. In a pilot NHP study, immunogenicity of RV-HIV viruses used as a prime or boost for DNA or NYVAC candidates was compared to a DNA prime/NYVAC boost benchmark scheme when administered together with adjuvanted gp120 protein. Similar neutralizing antibody titers, binding IgG titers measured against a broad panel of Env and Gag antigens, and ADCC responses were observed in the groups throughout the course of the study, and T cell responses were elicited. The entire data demonstrate that RV vectors have the potential as novel HIV-1 vaccine components for use in combination with other promising candidates to develop new effective vaccination strategies.

## Introduction

HIV transmission remains common with approximately 2 million new HIV infections occurring worldwide in 2017, underscoring the need for an effective vaccine^1^. The HIV-1 efficacy trial RV144 provided the first evidence that a HIV-1 vaccine is possible, where vaccination with ALVAC-HIV (vCP1521) in combination with subunit, alum-adjuvanted gp120 protein (AIDSVAX B/E) prevented HIV infection, with 60% and 31.2% efficacy documented at 12 months and 3.5 years, respectively^2^. This stimulated further exploration of new delivery systems and/or antigen designs and formulations for new, improved prime-boost regimens. In earlier studies, replication-deficient adenovirus, poxvirus, and DNA have been predominantly used in prime-boost combinations for the development of T-cell vaccine regimens^3,4^, while there is limited information on their use in combination with Env proteins to stimulate antibody responses. Therefore, the emphasis of current studies is on enhancing HIV-1 Env-specific humoral responses to increase the breadth, potency and durability of antibody responses, in addition to T cell responses through heterologous viral vector prime-boost regimens.

The RepliVax (RV) vaccine approach based on single-cycle flavivirus vectors attenuated by a deletion introduced in the gene(s) encoding viral structural proteins (C-prM-E) was initially applied to flavivirus targets, such as tick-borne encephalitis (TBE) virus, and subsequently to non-flavivirus targets^5–7^. Flaviviruses comprise a group of viruses with a positive-sense single open reading frame (ORF) RNA genome of ~11,000 nucleotides in length. The small enveloped viruses are transmitted by mosquitoes or ticks. From the point of view of vaccine development, they are of interest because flavivirus infection is known to elicit life-long homologous protective immunity, e.g., as exemplified by the characteristics of a prototype flavivirus live attenuated vaccine (LAV), yellow fever 17D (YF 17D) considered to be protective for life after single immunization. The single-cycle nature of RV vaccine constructs engineered for flavivirus targets are based on a capsid C gene deletion ensuring high attenuation *in vivo* of a vaccine candidate against flavivirus targets^6,7^. Inside infected cells, RV replicate like full flaviviruses which is expected to induce robust innate and adaptive responses^6,8^. We have reported earlier that RV flavivirus vaccine prototypes can match LAVs in terms of magnitude and durability of responses^6^. In addition, results in the NHP model indicated that a single dose of RV-TBE candidate should provide immunity against TBE of a higher duration compared to three complete doses of a human inactivated TBE vaccine^7^.

RV vaccine candidates against non-flavivirus targets are engineered to express an appropriate pathogen-specific immunogen(s) in place of large prM-E or C-prM-E deletions. They are propagated in helper cells expressing the C-prM-E cassette trans-complementing the vector deletion. We have expressed several immunogens from respiratory syncytial virus, influenza virus, and SIV in the West Nile (WN, NY99 strain) RV vector and demonstrated high attenuation and immunogenicity of the constructed recombinants in mice^9,10^. A single dose of a similarly constructed vaccine candidate against rabies (RV-Rabies G) was shown to protect dogs from rabies challenge two years post-immunization^9^.

In view of the potent immune responses and efficacy triggered by RV vectors, here we set out to assess in preclinical studies (mouse and NHP) the immunogenic capacity of the WN (NY99 strain) virus-based RV vector in the context of new heterologous HIV-1 prime/boost combination regimens. RV-HIV candidates expressing clade C Gag or Env (gp120TM) were constructed and their vaccine potential evaluated in *in vitro* and *in vivo* models, including NHPs in prime-boost combinations with recombinant DNA or the attenuated poxvirus NYVAC candidates expressing the same HIV-1 antigens as RV-HIV, and administered with adjuvanted subunit HIV-1 Env protein described previously^11,12^. Our findings revealed the potential benefit of the combination of RV/NYVAC/protein components as vaccination approach against HIV-1.

## Results

### Propagation of RepliVax-HIV variants in helper cells

**Figure 1:**
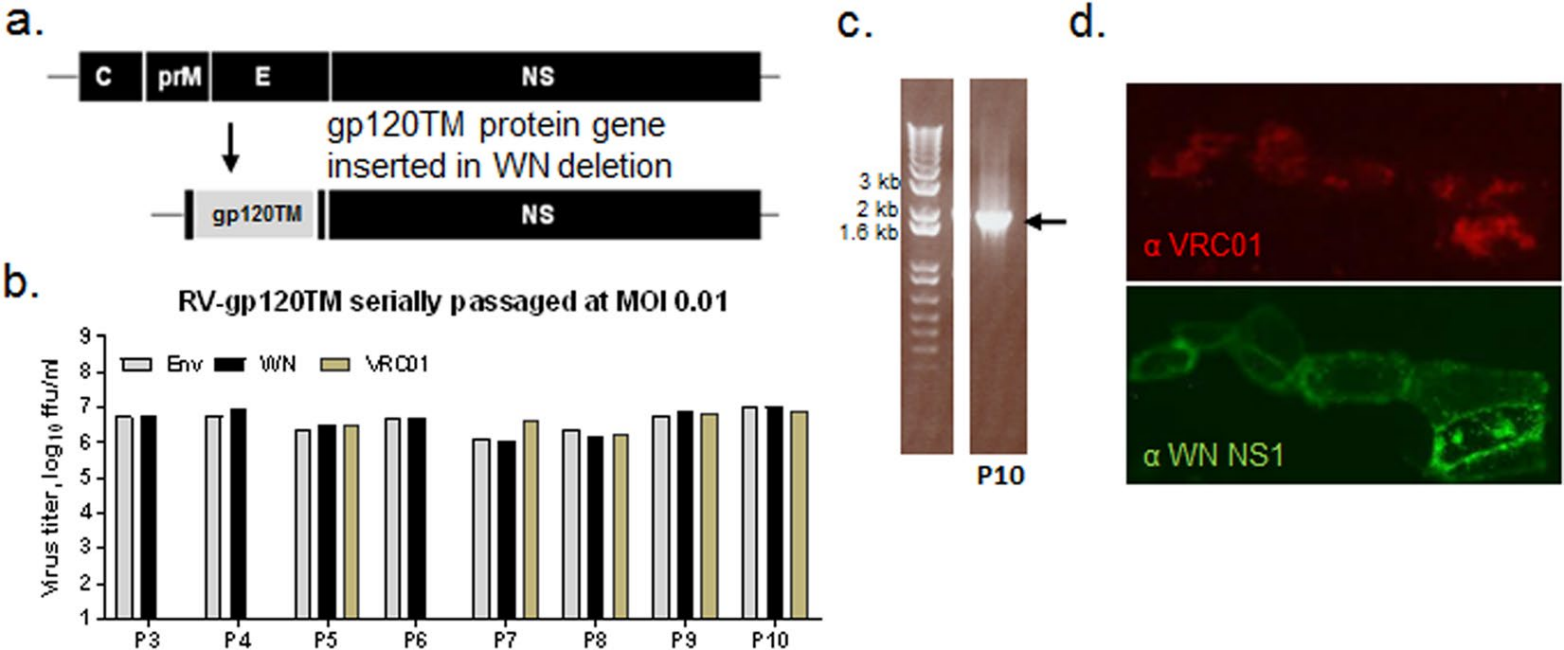
*In Vitro* replication and expression of RV-gp120TM. (**a**) Schematic of RV-gp120TM genome. The codon-optimized gp120TM (96ZM651) gene contains the native ZM96 signal sequence and FMDV 2A cleavage element at the N and C termini, respectively. (**b**) Maintenance of the gp120TM gene insert was assessed by 10 serial passages in helper Vero cells at MOI of 0.01. Viral titers were determined using anti-Env (goat α-gp120, Ab21179, Abcam) and anti-WN NS1 (Mab8152, Chemicon) antibodies providing titers of insert-containing and total infectious particles, respectively. VRC01 Mab was used to evaluate gp120TM conformation in the titration assay at selected passage samples 5, 7, 8, 9, and 10. (**c**) Stability of the gp120TM insert as evidenced by a single RT-PCR amplicon of the expected size produced from viral RNA isolated from P10 genetic stability passage using WN vector-specific primers outside of the insert. (**d**) Cell surface exposure and correct folding of gp120TM as shown by immunostaining of infected, formalin-fixed Vero cells using conformational VRC01 Mab; infected cells are also visualized with WN NS1-specific Mab 8152 (Chemicon).

RV-HIV recombinants were engineered to express clade C (strain 96ZM651, here termed ZM96) Env and Gag inserts in place of the C-prM-E deletion in the WN virus genome (Figs. 1A and S1A). Selected RV-gp120TM and RV-Gag candidates replicated efficiently in helper Vero cells expressing the WN virus C-prM-E proteins *in trans*, with titers exceeding 7 log_10_ ffu/ml. High stability of both Gag and Env (gp120TM) gene inserts was demonstrated during 10 serial passages in helper Vero cells at a controlled multiplicity of infection (MOI). Immunostaining of viral foci with HIV-1- and WN-specific antibodies showed equivalent titers of insert-containing virus and total infectious virus for all passages (Figs. 1B and S1B). RT-PCR and sequencing of the inserts in passaged samples confirmed the preservation of the full-length gene inserts (Figs. 1C and S1C). Efficient expression of the gene products, their cleavage from the vector virus polyprotein, and correct maturation and transport were confirmed by immunostaining of infected cells and Western blot analysis. Robust immunostaining was observed for the Env protein using the conformational human Mab, VRC01, indicating that gp120TM is properly folded to correctly present the CD4 receptor binding site (Fig. 1B,D). The Gag protein was efficiently secreted and protein size was as expected (Fig. S1D). The same Gag sequence expressed from a NYVAC vector generated HIV-like particles (VLPs)^13^. Other Env designs, specifically gp120, appeared to show lower levels of expression than gp120TM in the characterization assays and the longer Env variants, gp140 and gp140TM, were found to be less stable (data not shown).

### Attenuation in suckling mouse neurovirulence test

Newborn mice were inoculated intracranially with varying doses of the virus constructs (Table 1). RV constructs (RV-gp120TM, -Gag, and empty vector) were non-pathogenic in this model up to a dose of 10^6^ ffu, with 100% of the inoculated animals surviving. The NYVAC-KC-gp140 vaccine candidate based on a replication-competent NYVAC vector expressing clade C gp140 ZM96 was also highly attenuated, with mortality observed only at high doses (LD_50_ > 10^6^ pfu) consistent with previous data^14,15^. This was in contrast to wild-type vaccinia (Copenhagen strain) and the benchmark YF 17D controls (LD_50_ values ~ 10 and 0.5 pfu, respectively). This result demonstrates a very high attenuation, indicating that the RV-HIV vectors have a desirable safety profile.

**Table 1:** Attenuation of RV and NYVAC-KC vectors in suckling mice.

Age of mice	Construct	Dose(s) tested (log_10_ ffu/pfu)^a^	Mortality (%)	LD50 (log_10_ ffu/pfu)
2–3 days	RV-Gag	5, 6	0	N/A
	RV-gp120TM	5, 6	0	N/A
	RV-Empty	5, 6	0	N/A
	YF17D virus	0 −1 −2 −3	90 71 14 0	−1.13
	NYVAC-KC-gp140	8 7 6	96 87 33	6.3
	VC-2^b^	2 1 0	67 53 18	0.04

^a^Single dose IC inoculation of suckling CD1 mice; observation for 20 days.

^b^wild-type vaccinia strain Copenhagen (VC2).

### Immunogenicity in mice

**Figure 2:**
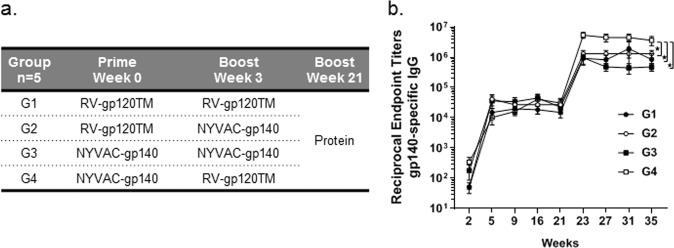
Binding IgG titers against clade C Env following immunization of mice with homologous or heterologous viral vector prime-boost combinations and a protein boost. (**A**) Female BALB/c mice were immunized i.m. at weeks 0 and 3 with either RV-gp120TM vector at 10^7^ ffu/dose or NYVAC-gp140 vector at 10^7^ pfu/dose and boosted at week 21 with 10 μg CN54 gp140 formulated in SMQ. (**B**) HIV-1 Env CN54 specific IgG titers were measured in serum collected at weeks 2, 5, 9, 16, 21, 23, 27, 31, and 35. Symbols represent the mean titers with SEM bars at each time point. *Denotes statistically significant group comparison at week 35 (2 Way ANOVA, Tukey, *P* < 0.05).

The RV-gp120TM vector administered in homologous or heterologous prime-boost combinations with NYVAC-gp140 vector elicited efficient antibody responses in mice (Fig. 2). Specific antibodies were detectable by ELISA as measured against CN54 gp140 protein in all groups at week 2 after the first dose. The binding IgG titers were significantly boosted following the second vaccination. The responses remained high until week 21 and were further increased by more than 50-fold by an additional protein boost at week 21 (10 μg CN54 gp140 adjuvanted with SMQ). Binding antibody titers elicited by group 4, NYVAC-gp140 prime followed by RV-gp120TM boost were significantly higher at week 35 than in the homologous prime-boost groups (G1 and G3) as well as the heterologous RV-gp120TM prime with NYVAC-gp140 boost (G2) (2-Way ANOVA, Tukey, *P* < 0.05) (Fig. 2).

**Figure 3:**
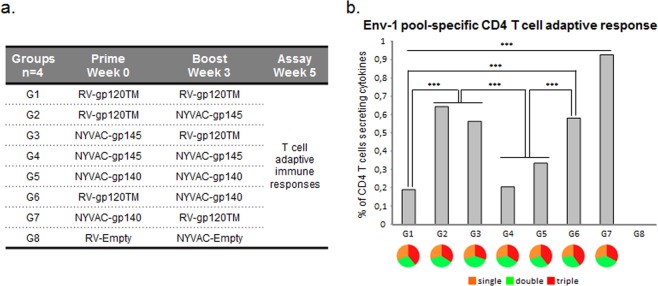
Adaptive HIV-1 Env-specific CD4 T cell immune response elicited by different homologous and heterologous combinations of RV and NYVAC vectors. (**A**) Female BALB/c mice were immunized i.m. at weeks 0 and 3 with either RV vectors at 10^7^ ffu/dose or NYVAC vectors at 10^7^ pfu/dose and spleens collected at week 5. (**B**) The magnitude and polyfunctionality of HIV-1-specific CD4 T cells were measured by ICS assay following stimulation of splenocytes derived from immunized animals with the ZM96gp140 peptide pool Env-1. The total value in each group represents the sum of the percentages of T cells secreting IFN-γ and/or IL-2 and/or TNF against Env-1 peptide pool. All data are background-subtracted. ***P < 0.001. *P* values indicate significantly higher responses between groups. Functional profiles are grouped and colour-coded based on the number of functions.

In a separate mouse study, polyfunctional HIV-1 Env-specific CD4 T cell responses were assessed in mice immunized with different vector prime-boost regimens in the absence of adjuvanted protein (Fig. 3A). HIV-1-specific T cell responses were detected in all groups, except empty vector controls, at week 5 (2 weeks post the second vaccination). All immunized groups showed similar ratios of T cells secreting pro-inflammatory cytokines IFN-γ and/or IL-2 and/or TNF (Fig. 3B). Interestingly, mice immunized with homologous vector vaccine regimen showed the lowest CD4 T cell responses whereas the heterologous prime-boost combinations induced significantly higher CD4 responses (p < 0.001) (Fig. 3B). The heterologous NYVAC-gp140 prime followed by RV-gp120TM boost (**group 7**, Fig. 3) showed a significantly higher CD4 T cell responses than all other groups (*P* < 0.001), which was in accordance with the Env binding antibody data in the first mouse study.

### Assessment of immunogenicity of prime-boost combinations in NHP

Since the heterologous prime-boost regimens proved superior in mice, we further tested in the nonhuman primate model the immunological effect of RV in combination with either DNA or NYVAC vectors that express Env or Gag-Pol-Nef. The immunization schedule and vaccine formulations are summarized in Table 2. All groups received in parallel adjuvanted gp120 proteins (TV and 1086) inoculated into a separate site at immunization weeks 0, 4, 12, and 24, as a modification from the RV144 trial^2^, to evaluate the impact of the different viral vectors on HIV-1 Env-specific antibody responses. Group 1 receiving the DNA prime (weeks 0 and 4) and NYVAC boost (weeks 12 and 24) served as an immunological benchmark as previous studies have demonstrated that the DNA prime - NYVAC boost sequence enhances HIV-1-specific immune responses to higher levels than NYVAC vector regimen alone^4,11^. All vaccine formulations were well-tolerated, with no adverse events observed in any of the groups throughout the course of the study. Vaccine regimens were serologically assessed for the magnitude, breadth, and durability of the binding and functional antibody titers and T cell responses at weeks 0, 6, 14, 26, and 36 as outlined in the immunization schedule (Table 2).

**Table 2:** Nonhuman primate study design.

Group	Construct	Total dose^a^	Immunization weeks	Sample collection weeks
1. DNA/NYVAC	**DNA:** Gag (ZM96)-PolNef (CN54) + **DNA:** Envgp140 (ZM96)	4 mg	0 & 4	0, 6, 14, 26, & 36
	**Protein:** TV1 gp120 & 1086 gp120	0.1 mg	0, 4, 12, & 24	
	**NYVAC:** Envgp140(ZM96) + **NYVAC:** Gag (ZM96)PolNef (CN54)	1 × 10^8^ pfu	12 & 24	
2. DNA/RV	**DNA:** Gag (ZM96)-PolNef (CN54) + **DNA:** Envgp140 (ZM96)	4 mg	0 & 4	0, 6, 14, 26, & 36
	**Protein:** TV1 gp120 & 1086 gp120	0.1 mg	0, 4, 12, & 24	
	**RV:** Envgp120TM (ZM96) + **RV:** Gag (ZM96)	1 × 10^8^ ffu	12 & 24	
3. RV/NYVAC	**RV:** Envgp120TM (ZM96) + **RV:** Gag (ZM96)	1 × 10^8^ ffu	0 & 4	0, 6, 14, 26, & 36
	**Protein:** TV1 gp120 & 1086 gp120	0.1 mg	0, 4, 12, & 24	
	**NYVAC:** Envgp140 (ZM96) + **NYVAC:** Gag (ZM96)PolNef (CN54)	1 × 10^8^ pfu	12 & 24	
4. NYVAC/RV	**NYVAC:** Envgp140 (ZM96) + **NYVAC:** Gag (ZM96)PolNef (CN54)	1 × 10^8^ pfu	0 & 4	0, 6, 14, 26, & 36
	**Protein:** TV1 gp120 & 1086 gp120	0.1 mg	0, 4, 12, & 24	
	**RV:** Envgp120TM (ZM96) + **RV:** Gag (ZM96)	1 × 10^8^ ffu	12 & 24	

^a^All groups IM immunization at indicated time points; Proteins formulated with MF59 prior to immunization.

**Figure 4:**
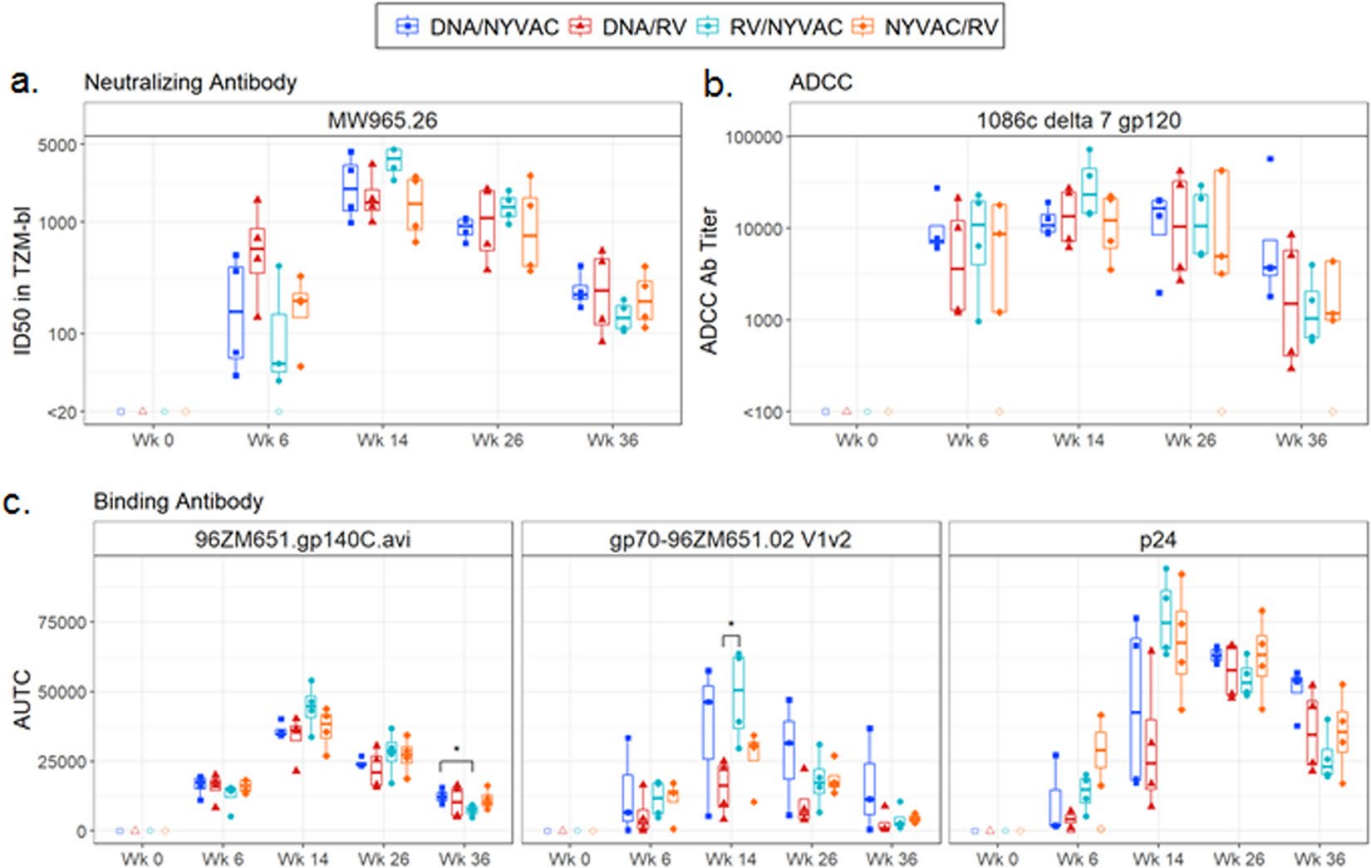
Humoral immune responses of the different vaccine regimens in Rhesus Macaques. (**A**) Neutralization titers were measured in sera at the indicated time points against clade C HIV-1 virus (MW965.26, tier 1A) using the TZM-bl assay. (**B**) ADCC-mediated antibody responses were measured using GranToxiLux (GTL) assay using the 1086.c gp120 antigen. (**C**) Magnitude of binding IgG titers were measured by BAMA expressed as the AUTC against homologous 96ZM651 antigens gp140, Gag p24 and V1/V2. Each dot represents the value for one immunized macaque. Box plots show the response distribution among positive responders only. The box indicates the median and interquartile range (IQR); whiskers extend to the furthest point within 1.5 times the IQR from the upper or lower quartile. *Denotes statistically significant group comparison (Wilcoxon rank-sum, *P* < 0.05). No significant results were observed after multiple test correction.

Vaccine-induced functional neutralizing antibody responses were observed in all groups as measured with the TZM-bl cell assay against the tier 1a clade C viral isolate MW965.26 (Fig. 4A). Generally, animals had peak responses at week 14 (after 3^rd^ immunization) instead of week 26 (post 4^th^ immunization). However, the differences between these time-points were not significant. No significant differences in the magnitude of neutralizing antibody titers were observed between the different vaccine regimens at any of the time-points analyzed. There were no positive responders against viral isolates 96ZM651.02, TV1.21, and C1080.c03 in any of the vaccine regimens including the benchmark Group 1 (data not shown).

Functional antibodies dependent on Fc receptor engagement for cell-mediated immunity were evaluated by the GranToxLux (GTL) and Luciferase (Luc) antibody-dependent cell-mediated cytotoxicity (ADCC) assays. All groups exhibited positive ADCC-mediated antibody responses against clade C HIV-1 gp120 from isolate 1086c, that is homologous to the adjuvanted protein administered at all time-points in all groups (Fig. 4B). Peak titers were observed at week 14, with Group 3 (RV/NYVAC) trending the highest, however, differences between groups were not significant. Generally, the magnitude of titers elicited post 2^nd^ immunization in all groups at week 6 were comparable to that observed after the 3^rd^ and 4^th^ immunizations, suggesting that the ADCC responses reached their maximum early during the immunization schemes. The overall kinetics profile of the granzyme B activity to the ADCC antibody titer observed between the groups and time-points were similar, with some differences in extent, against isolates 1086c, 96ZM651 (not shown) and TV1 (Fig. S2). All groups showed 100% response rates for all antigens specifically groups 1 and 3 at all-time points, whereas groups 2 and 4 showed lower rates at the later time points (Fig. S2). Similar to neutralizing titers, all groups including the benchmark control showed a decline in ADCC Ab titers at week 36, 12 weeks post the final boost with vectors and adjuvanted proteins.

Serum IgG binding antibodies were measured at weeks 0, 6, 14, 26, and 36 in all groups to assess the magnitude and breadth of responses against Env gp120, gp140, and murine leukemia virus (MuLV) gp70 scaffold V1/V2 and V3 proteins from a multi-clade envelope panel, as well as gp41 and p24 (Gag). Hundred percent seroconversion was observed against all gp120/140 readout antigens tested from the different clades at all time-points post 2 immunizations (week 6), demonstrating that all vaccine regimens can induce cross-reactive antibodies in addition to 96ZM651 gp140, the homologous clade Env expressed from the viral vectors and DNA (Figs. 4C and S3). All groups showed similar antibody kinetics where peak titers were observed at week 14 and declining at week 36, including the Group 1 benchmark control (Figs. 4C and S4). Moreover, Groups 1 and 3 showed 100% seroconversion against all V1/V2 and V3 test antigens in the test panel at week 6 through week 36, whereas the other groups showed some variation in epitope recognition between clades at the tested time-points (Figs. 4C and S5). As expected, lower responses to gp41 were detected in groups immunized with the RV-gp120TM variant as the construct did not contain this region, and limited seroconversion was observed between the different time-points with the NYVAC-gp140 construct (Fig. S3). All groups showed a 100% seroconversion at week 14 post three immunizations showing that boosting with heterologous vectors, specifically in Groups 3 and 4, can significantly enhance immunity (Figs. 4C and S3). Both serum and rectal mucosal IgA titers were low in all groups, while rectal mucosal IgG titers were readily detectable with titers peaking at week 14 like that observed for the other assay parameters (data not shown).

**Figure 5:**
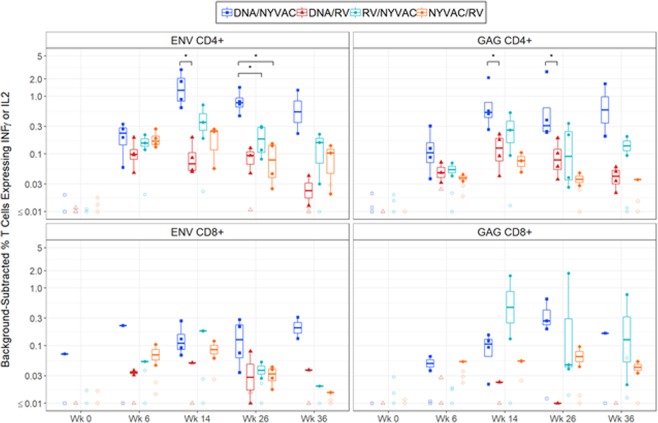
HIV-1-specific CD4 and CD8 T cell immune responses in Rhesus Macaques. Frequency of memory CD4^+^ and CD8^+^ T–cell responses were measured by the ICS assay for IFN-γ and/or IL-2 cytokine markers following stimulation of PBMCs with HIV-1 Env and Gag peptide pools. The percent positive T cell measurement was background-adjusted by subtracting negative controls. Each dot represents the value for one immunized macaque. Box plots show the response distribution among positive responders only. The box indicates the median and interquartile range (IQR); whiskers extend to the furthest point within 1.5 times the IQR from the upper or lower quartile. *denotes statistically significant group comparison (Wilcoxon rank-sum, *P* < 0.05).

The magnitudes of the HIV-1-specific T cell immune responses induced by the different vaccine regimens were analyzed by intracellular cytokine staining (ICS) assay. Efficient IFN-γ and/or IL-2 responses were observed among CD4^+^ and CD8^+^ memory T cells following stimulation with peptide pools that spanned the HIV-1 Env, Gag, Pol, and Nef antigens present in the inserts for all groups (Fig. 5). The magnitude of HIV-1 Env- and Gag-specific CD4 and CD8 responses were highest in Groups 1 and 3 (Fig. 5). Group 2, DNA prime with RV boost, showed significantly lower HIV-1 Env and Gag specific CD4 responses than Group 1, DNA prime followed by NYVAC boost at week 14 and 26. Similarly, HIV-1 Env- and Gag-specific CD8 responses were the lowest for Group 2 suggesting that this DNA/RV vector regimen is suboptimal. Groups 3 and 4 receiving the viral vector prime-boost regimens showed peak titers trending at week 14 similarly to the other immune read outs shown.

### *Ex vivo* immunogenicity in human monocytes and dendritic cells

**Figure 6:**
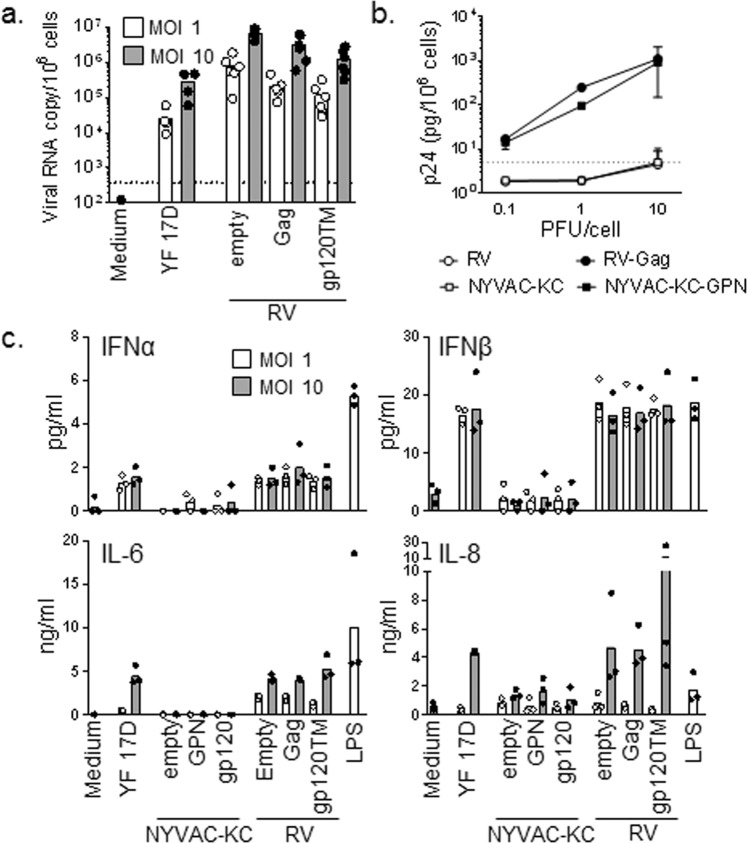
Transduction efficiency and cytokine production by human monocytes. Monocytes from healthy volunteers were infected for 24 h with YF 17D, NYVAC-KC, NYVAC-KC-GPN, NYVAC-KC-gp120, RV-Gag or RV-gp120TM (white bars: MOI 1; grey bars: MOI 10). (**A**) YF17D and RV RNA copies were quantified by RT-PCR. (**B**) p24 concentrations in cell culture supernatant were measured by electroluminescence. (**C**) IFNα, IFNβ, IL-6 and IL-8 concentrations in cell culture supernatants were measured by Luminex. LPS (10 ng/ml) was used as a positive control. Dots and bars represent individual data and mean, respectively.

*Ex vivo* experiments utilizing human monocytes demonstrated that the single-cycle RV vectors transduced cells at least as efficiently as the replication competent YF 17D vaccine control virus as shown by quantification of viral RNA copies per cell (Fig. 6A). The level of p24 production was similar at the different multiplicities of infection (0.1, 1, and 10) between the NYVAC-KC-GPN and RV-Gag vectors post-transduction (Fig. 6B). Interestingly, the RV variants stimulated cytokine (IFNα, IFNβ, IL-6, and IL-8) production by human monocytes similarly to that of YF 17D while the pattern was different from NYVAC-KC variants (Fig. 6C). Similar effect as for NYVAC-KC was observed with the non-replicating NYVAC vector (data not shown). In addition to these studies, CD8 T cells from HIV-1-infected individuals were evaluated in a Gag-specific recall response upon stimulation with either NYVAC-GPN or RV-Gag viruses compared to controls (Gag peptide and SEB). Both RV-Gag and NYVAC-GPN efficiently stimulated Gag-specific CD8 recall response to similar levels as the control groups (Fig. S6). In summary, *ex vivo* studies show that both vectors express efficiently post transduction, elicit similar recall responses, and stimulate different cytokine profiles which could be beneficial in a heterologous prime-boost vector vaccine regimen.

## Discussion

The RV144 trial has been one of the most successful trials showing that a HIV vaccine is possible in preventing infection and efforts continue with improving the priming and boosting components for a vaccine. Current challenges with developing an effective HIV vaccine include the need to understand the critical cellular responses, the level of protective antibodies required, and the balance between the cellular and humoral responses to control and prevent HIV infection^16–18^. For HIV-1 clade C, there are ongoing phase IIb efficacy trials in South Africa with the combination of a poxvirus (ALVAC plus gp120 protein; https://www.clinicaltrials.gov/ct2/show/NCT02968849) or adenovirus 26 mosaic sequences followed by trimeric gp140 boost (HVTN 705; https://www.clinicaltrials.gov/ct2/show/NCT03060629). These efficacy trials have in common the use of a viral vector (either a recombinant poxvirus or adenovirus) as a priming component and Env protein (gp120 or gp140) as the booster component. Thus far, the only correlates of protection, defined largely from the RV144 trial, are the induction of non-neutralizing antibodies, occurrence of anti-V1/V2 binding antibodies, induction of IgG3 binding antibodies, T cell responses and reduced anti-IgA antibodies^19–24^. Different prime-boost regimens with heterologous viral vectors may be an efficient strategy for stimulating multiple arms of adaptive and innate immunity as the vector backbone inherently acts as a potent adjuvant, different vectors trigger unique responses, and heterologous regimen limits anti-vector immunity^16^. With the aim to explore how a novel flavivirus vector RV-HIV could impact on the immune responses to HIV-1 antigens, we have performed preclinical studies in mice and in NHP immunized with the RV vectors in combination with a poxvirus vector NYVAC or DNA expressing the same HIV antigens, together with Env (gp120) protein component in MF59 adjuvant. We then explored the immune responses towards HIV-1 clade C immunogens Env and Gag by evaluating B cell (antibodies, IgG, V1/V2, cross-reactivity, neutralizing, ADCC responses) and T cells (CD4, CD8, recall responses, transduction efficiency, cytokines), delivered by heterologous and homologous vaccine combinations.

The RV platform was developed to take advantage of the potent immunogenicity triggered by the flavivirus vectors as demonstrated with the yellow fever live attenuated vaccine (LAV), YF 17D, inducing long-lasting immunity after a single vaccine dose^25^. To assess the potential of RV vectors as vaccine candidates against HIV-1, in this investigation we generated RV-based recombinants expressing HIV-1 Env (gp120TM, similar construct as used in the RV144 clinical trial but from clade C), and Gag (VLP formation as previously described from a NYVAC vector^13^) and studied their immunogenicity profile in animal models (mouse and NHP). The data in this study confirm our previous observations that RV vectors can be engineered to stably deliver large non-flavivirus antigens^9,10^, specifically gp120TM and Gag of an HIV-1 clade C virus, with high production yields in trans-complementing Vero cell substrate suitable for manufacturing. It should be pointed out that RV vectors expressing the longer Env gene cassettes, gp140 or gp140TM from strain 96ZM651, were unstable possibly due to sequence-specific toxicity to the Vero cell, as stability can vary between Env sequences and depending on the length of gene cassettes which has proven challenging when expressing from other viral vectors^26–28^. Hence, studies were conducted with RV-gp120TM and –Gag vectors, both being highly stable. The recombinant vectors efficiently expressed HIV-1 antigens *in vitro* and the Env protein was presented at the cell surface and was recognized by a monoclonal antibody that binds to the gp120 epitope that interacts with the HIV-1 CD4 binding site^29^, suggesting that the expressed protein maintained correct folding.

As expected, the single-cycle RV-HIV constructs were shown to be avirulent in the neonatal mice, findings analogous to our previously published data showing the high safety profile of RV as compared to the licensed YF 17D vaccine^9,10^. The immunogenicity studies in the mouse model demonstrated that the recombinant RV-HIV vectors were well-tolerated following peripheral inoculation, indicative of an acceptable safety profile. In addition, no severe adverse events were observed during the NHP study, where animals were scored for clinical symptoms, general behavior, body weight, temperature, local reactions, clinical chemistry and hematology by the attending veterinarian. Our data demonstrate that prime-boosting with heterologous viral vector formulations (RV and NYVAC) with DNA and adjuvanted protein may be a well-tolerated vaccination approach.

Different prime-boost regimens with heterologous viral vectors could be an efficient strategy to maximize immune responses for improved HIV vaccines. Poxviruses have been studied extensively as HIV vaccine candidates and shown to elicit polyfunctional T cell responses with acceptable safety profile^16^. The highly effective YF 17D flavivirus vaccine elicits persistent neutralizing antibodies and memory T cells and is one rationale for generating recombinant RV-HIV vectors^30^. This study is the first to explore the immune responses towards HIV-1 clade C immunogens Env and Gag delivered by heterologous vaccine combinations of poxvirus and flavivirus vectors to take advantage of the unique immune responses each vector elicits. Administration of the homologous prime-boost NYVAC-gp140 or RV-gp120TM in mice elicited comparable levels of binding antibodies as the heterologous groups and significant boosting effect was observed for all groups suggesting anti-vector immunity had little impact on antibody stimulation. Protein boost of mice primed with NYVAC and boosted with RV showed a higher HIV-1 Env-specific binding antibody response compared to the other schedules. In a separate mouse study, it was also shown that the HIV-1-specific CD4 T cell responses were significantly higher for the heterologous NYVAC- RV schedule than the homologous prime-boost combinations. It is not clear from this one mouse study if there is an advantage to prime with NYVAC and boost with RV or vice versa due to differences in the expressed Env cassettes, however the data shows the highest response rates with NYVAC-gp140 prime followed by RV-gp120TM boost. The HIV-1-specific CD4 T cell responses were polyfunctional for all groups. Collectively the mouse data suggests improved immune responses using a heterologous prime-boost vaccination strategy over homologous, however a more in-depth study may be required to better understand the value of the heterologous versus homologous prime boost.

Based on the findings in mice, we next performed a NHP study, in which immunogenicity of RV-HIV constructs administered in heterologous prime-boost combinations with other vaccine candidates was assessed in comparison to recombinant DNA prime - NYVAC boost vaccination used as a benchmark control. Overall, the study demonstrated that RV-HIV variants can be as immunogenic as DNA and NYVAC vaccine candidates when co-administered with MF59 adjuvanted gp120 protein with respect to the magnitude and breadth of binding antibody responses, neutralizing antibody titers, ADCC, and T cell responses. We observed that RV vectors in combination with DNA or NYVAC were strong immunogens able to induce a broad spectrum of HIV-1-specific B and T cell immune responses that is believed to be associated with immune efficacy as learned from the RV144 phase III clinical trial^19–24^. Although the heterologous vector combination of RV with DNA or NYVAC induced good immune responses, at the peak of the response (week 14) there was a trend to higher levels of HIV-1-specific IgG titers, neutralizing antibodies and ADCC elicited by the combined RV/NYVAC vectors. Efficacy studies in the NHP model have demonstrated the significance of humoral responses in protecting against infection^31–33^, hence the robust activation of immune markers induced by RV in combination with DNA or NYVAC highlight the utility of this RV platform. This is remarkable given the inability of the virus to replicate and cause spreading infection, thus the overall similarity of immunity elicited by RV is promising.

RV-HIV constructs were also found to stimulate innate responses *ex vivo* in human monocytes/DCs to levels comparable to YF 17D, and to elicit robust recall HIV-1-specific CD8 responses. The similarity of the cytokine signatures of the single-cycle RV constructs to the replication competent YF 17D vaccine, an extremely strong human immunogen, suggests that a robust and durable immunity could be induced by non-replicating RV vectors. The mechanisms for long term immunity warranted by a replication competent YF 17D virus is not fully understood and further evaluations of the RV vector to YF 17D may help to elucidate the immune characteristics.

Based on the selective activation of immune markers, some of which have been correlated with reduced risk of infection^19–24^, our study highlights the benefit of RV, NYVAC and protein components as a combined immunization approach against HIV-1. Moreover, the NHP study showed that addition of the protein component at all immunization time points (0, 4, 12, 24, 34 weeks) with the different components was an efficient way to enhance the levels and breadth of HIV-1 specific B and T cell responses, as well as durability of these immune responses. In general, there was a trend of better immune responses with the combination of RV/NYVAC/protein, with the protein always added during immunization, a protocol that accelerates the induction of HIV-1 specific immune responses. This faster development of HIV-1 humoral immune responses has been confirmed in a prophylactic phase 1b clinical trial with gp120 protein added together with NYVAC or DNA vectors expressing the same HIV-1 antigens as in this work^34^.

In summary, the study presented here is the first to evaluate immune responses induced by a heterologous prime-boost vaccination strategy utilizing poxvirus and flavivirus vectors expressing HIV-1 immunogens and show that this immunization strategy can enhance the levels of binding and neutralizing antibody titers and ADCC responses. While there are several immunogenicity trials ongoing with other viral vectors (HVTN-Ongoing-Trials 2019), collectively, our data indicate that RV is an attractive vector for the HIV vaccine field to enhance immune responses, and the described constructs could be promising candidates for a vaccine for sub-Saharan Africa (Clade C virus). The data validate further development of heterologous prime-boost strategies including evaluation in the NHP challenge model and/or humans to assess vaccine efficacy. Furthermore, the poxvirus and flavivirus vector heterologous prime-boost vaccination strategy could be tested for other viral candidates and a full spectrum of immune responses assessed like done in this study.

## Materials and Methods

### Ethics statement

The animal studies performed at Arizona State University (ASU) were approved by the Institutional Animal Care and Use Committee at ASU in accordance with all federal regulations and guidelines. The animal studies conducted at Fred Hutchinson Cancer Research Center (FHCRC) were approved by the FHCRC’s institutional animal care and use committee. The animal studies performed at Centro Nacional de Biotecnología (CNB-CSIC, Madrid, Spain) were approved by the Ethical Committee of Animal Experimentation (CEEA) at CNB in accordance with International EU Guidelines 2010/63/UE on protection of animals used for experimentation and other scientific purposes, Spanish National Royal Decree RD 53/2013 and Spanish National Law 32/2007 on animal welfare, exploitation, transport and sacrifice (permit number PROEX 014/15). The rhesus macaque study was carried out in strict compliance of “Guide for the Care and Use of Laboratory Animals” of DHHS (NIH 85-23). Protocol was approved by the Institutional Animal Care and Use Committee of Bioqual, Inc.

### Construction of viral vectors expressing HIV-1 genes

RepliVax-HIV variants based on the West Nile Texas 2003 virus backbone were constructed using the coding sequences of gp120TM and Gag genes of HIV-1 strain 96ZM651 (here termed ZM96) (Table S1). HIV-1 gene cassettes were adapted to human codon usage, direct nt sequence repeats of ≥9nts were removed by silent nt substitutions, and expression was enhanced (balanced GC-content and avoidance of RNA secondary structures) using the GeneOptimizer algorithm^35^. At the N-termini, the gp120TM cassette contains the native 96ZM651 Env signal peptide and amino acids encoding gp120 (33–516) fused to the transmembrane domain of 96ZM651. The Gag cassette contains ubiquitin autoprotease to free the N-terminal Gly necessary for Gag myristoylation and secretion. Both cassettes contain a foot-and-mouth disease virus self-cleavage element 2a (FMDV2a) at the C-terminus. The cassettes were cloned in place of the C-prM-E deletion in the WN vector virus downstream from the first 31 codons of the C protein containing the WN virus RNA cyclization element like previously published^9,10^. RV-gp120TM and RV–Gag viruses were recovered following transfection of helper Vero cells with *in vitro* RNA transcripts synthesized using T7 RNA polymerase or plasmid DNAs engineered to contain the eukaryotic cytomegalovirus (CMV) major immediate-early-1 promoter to launch the viral genome, and the viruses were further propagated in helper Vero cells.

The NYVAC and NYVAC-KC viral vectors used in the studies included the genetically attenuated VACV-based vector NYVAC-WT (vP866, provided by Sanofi-Pasteur) and the recombinants NYVAC-gp140, expressing a soluble trimeric gp140 from HIV-1 clade C 96ZM651 (here termed ZM96 and sequences are common to the shorter gp120TM variant used in RV)^13^, NYVAC-gp145, expressing a membrane-bound trimeric gp140 from HIV-1 clade C ZM96 (submitted manuscript) and NYVAC-GPN, expressing Gag (from clade C ZM96) - Pol - Nef (from clade C CN54) as VLPs (Table S1)^13,15^.

The plasmid transfer vectors plZAW1-gp140 (ZM96), plZAW1-gp140TM (ZM96) and plZAW1-Gag (ZM96)-Pol-Nef (CN54) were constructed as previously described^13^. Infection/transfection procedure, recovery of the NYVAC-HIV variant viruses using the β-Gal selection marker, plaque purification steps and growth in African green monkey kidney cells (BSC-40) were as previously described^13^.

### Cells and viruses

Helper Vero cells for propagation of RepliVax-HIV variants were generated by transfecting Vero cells with the Venezuelan equine encephalitis virus replicon (rVEE, based on the TC-83 vaccine strain) expressing the WN virus C-prM-E genes and a puromycin N-acetyl-transferase selective marker constructed similarly to rVEE-WN C helper replicon^5,36^. The Vero helper cells (VeroA2) were cloned and propagated in FBS-containing MEM medium supplemented with 10 μg/ml of puromycin. Propagation of RV-HIV variants in Vero helper cells and generation of serum free viral stocks for animal studies and *in vitro* characterization assays including viral titration were done as previously described^7,9,10^. Briefly, helper Vero cells were infected at MOI 0.01 and overlaid with FBS (2%)-containing medium, at 24–48 hours post-infection medium was removed, cells were washed with PBS, and overlaid with non-FBS-containing medium. Supernatants were harvested 72–96 hours post-infection, concentrated (Centricon Plus-70 centrifugal filter unit, Amicon) supplemented with sorbital (10% final concentration) and stored at −80 °C. Infectious titers determined by immuno-focus assay in Vero cells, as described previously^6^, using antibodies against either Envelope (Abcam#21179) or Gag (Abcam#63917) and WN NS1 (Mab8152; Millipore). Primary antibodies were detected with either anti-goat IgG-HRP or anti-rabbit IgG-HRP (Thermo/Pierce) or anti-mouse IgM-HRP (Millipore) conjugate antibodies. Titers were calculated by counting WN NS1-and Env- or Gag- stained cells and expressed as focus forming units (ffu/ml). In addition, colocalization and infectious titer was further confirmed in the immune-focus assay utilizing secondary fluorescent conjugate antibodies, anti-goat IgG 594 (Env) with anti-mouse IgM-FITC (WN NS1) or anti-rabbit IgG 488 (Gag) with anti-mouse IgM-594 (WN NS1) (Thermo/Pierce). Detection of gp120TM protein on infected Vero cells was performed 48 hours post-infection by 4% paraformaldehyde fixation and probing with Human VRCO1 (kindly provided by Gary Nabel’s lab) and WN NS1 (Mab8152; Millipore) antibodies and primary antibodies detected with either anti-human IgG-594 or anti-mouse-FITC (Thermo/Pierce). The predicted protein size of Gag was determined by Western Blot analysis by running harvest supernatant or concentrated supernatant day 3–4 post infection on a 4–12% Bolt Bis-Tris gel, transferred to a nitrocellulose blot, and probed with anti-Gag (Abcam#63917, 1:1000) specific antibody.

NYVAC-based recombinant viruses were grown in BSC-40 cells and amplified in primary chicken embryo fibroblast (CEF) cells. Both cell lines were grown in Dulbecco’s modified Eagle’s medium (DMEM) supplemented with 100 units/ml of penicillin, 100 µg/ml of streptomycin and 10% newborn calf serum (NCS) for BSC-40 or 10% fetal calf serum (FCS) for CEF cells. NYVAC infections were performed with 2% NCS or FCS. Amplification of NYVAC recombinant viruses in CEF cells was followed by virus purification through two 36% (w/v) sucrose cushions to generate stocks for animal studies as previously described^13^.

### Neurovirulence assay in newborn mice

For studies in newborn mice, pregnant CD1 mice were purchased from Charles River Laboratories at approximately 10 days gestation. The animals were housed one animal per cage. Intracranial infections with the viruses indicated in Table 1, using a total volume of 10 µL, were conducted at 48 to 72 hours post-birth of the pups (at least 10 pups per virus), using a 27-gauge needle, as previously described^37^. Animals were monitored twice daily for 20 days for morbidity and mortality.

### Mouse immunization studies

For the analysis of the HIV-1 Env-specific humoral immune response, female BALB/c mice, 5 to 6 weeks old were purchased from Harlan Laboratories (Indianapolis, IN) and maintained in the Fred Hutchinson Cancer Research Center (FHCRC) small animal facility. Mice were divided into 4 test groups with 5 animals per group. The study schema is depicted in Fig. 2A. At week 0, animals were injected with 10^7^ ffu of RepliVax ZM96gp120TM or 10^7^ pfu of NYVAC-ZM96gp140. At week 3, groups received either a homologous or heterologous boost at the same dose. At week 21, all mice received 10 µg CN54 gp140 protein (Polymun, Vienna, Austria) formulated with SMQ, a squalene-in-water emulsion containing cholesterol, MPL and QS21. SMQ was provided by the Vaccine Formulation Lab at the University of Lausanne. All intramuscular injections were performed within 1 hour of formulation in the rear quadriceps using a 28-gauge insulin syringe. Serum was collected from mice at weeks 2, 5, 9, 16, 21, 23, 27, 31 and 35 for enzyme-linked immunosorbent assay (ELISA) to measure the humoral adaptive immune response against HIV-1 envelope antigen.

For the analysis of the HIV-1 Env-specific T cell immune response, groups of female BALB/c mice (n = 4, 6 weeks old) purchased from Charles River Laboratories were housed at CNB-CSIC animal facility and immunized with 1 × 10^7^ pfu of NYVAC-gp140 or NYVAC-gp145 or 1 × 10^7^ ffu of RV-WN or RV-gp120TM. Three weeks later, animals were immunized with NYVAC or RepliVax constructions as in the priming and 2 weeks after the last immunization, mice were sacrificed and spleens processed for Intracellular Cytokine Staining (ICS) assay to measure the cellular adaptive immune responses against HIV-1 antigen. The immunization schedule is depicted in Fig. 3A.

### Antibody measurement by ELISA

ELISA’s were performed following standard procedure. 2HB plates (96-well; Nalgene Nunc, Rochester, NY) were coated overnight with 90 ng/well HIV-1 Env-CN54 protein, diluted in 1X coating buffer (Cat # 6247ImmunoChemistry Technologies, Bloomington, MN), then washed with buffer (150 mM NaCl, 0.1% Tween-20), blocked for 1 hour in 5% nonfat milk, 3% heat-inactivated goat serum and 0.2% Tween-20 in PBS and then washed again. Serum samples were serially diluted in blocking buffer, and then added to the wells in duplicate and incubated for 2 hours at 37 °C. The plates were then washed and HRP-conjugated anti-mouse IgG (Thermo Scientific) was added at 1:2500 dilution and incubated for 1 hour at 37 °C. After washing, the plates were developed with 3,3′–5,5′-tetramethylbenzene (BM Blue POD substrate; Roche Applied Science, Indianapolis, IN); the reaction was stopped by addition of 1M H_2_SO_4_ and the optical density was read at 450 nm using a SpectraMax M2 plate reader (Molecular Devices, Sunnyvale, Ca). The reported titers correspond to the reciprocal of the highest serum dilution showing a three times higher OD value than background.

### Mouse ICS assay

The HIV-1 ZM96 gp140 peptides, provided by the Centralised Facility for AIDS Reagents (NIBSC, UK) and spanning HIV-1 gp140 from clade C (ZM96) included in the recombinant viruses NYVAC-gp140 and NYVAC-gp145, were pooled. Env-1 pool has been reported to be the most immunogenic combination and was therefore selected as stimulus in the following studies^13^. For the ICS assay, 4 × 10^6^ splenocytes from immunized mice (depleted of red blood cells) were seeded on 96-well plates and stimulated for 6 hours in complete RPMI 1640 (100 units/ml of penicillin/100 μg/ml of streptomycin, 2 mM L-glutamine, 10 mM Hepes and 0.01 mM β-mercaptoethanol) with 10% FCS and containing 1 µl/ml Golgiplug (BD Biosciences), anti-CD107a-Alexa 488 (BD Biosciences) and 5 μg/ml of HIV-1 Env-1 pool. At the end of the stimulation period, cells were washed, stained for the surface markers, fixed and permeabilized (Cytofix/Cytoperm Kit; BD Biosciences) and stained intracellularly using the appropriate fluorochrome-conjugated antibodies. Dead cells were excluded from the analysis using the violet LIVE/DEAD stain kit (Invitrogen). For the analysis of CD4 and CD8 T cell responses, the following fluorochrome-conjugated antibodies were used: CD3-PE-CF594, CD4-APC-Cy7, CD8-V500, CD127-PerCPCy5.5 and CD62L-Alexa700 for phenotypic analyses and IL-2-APC, IFN-γ-PE-Cy7 and TNF-α-PE for functional analyses. All antibodies were from BD Biosciences. Cells were acquired using a GALLIOS flow cytometer (Beckman Coulter). Analyses of the data were performed using the FlowJo software version 8.5.3 (Tree Star, Ashland, OR). The number of lymphocyte-gated events ranged between 1 × 10^5^ and 1 × 10^6^. After gating, Boolean combinations of single functional gates were then created using FlowJo software to determine the frequency of each response based on all possible combinations of cytokine expression or all possible combinations of differentiation marker expression. For each population, background responses detected in the non-stimulated control samples were subtracted from those detected in stimulated samples for every specific functional combination and the percentages of cells producing cytokines obtained in the control populations were also subtracted in all the groups in order to remove the non-specific responses detected as background.

### Rhesus macaque study

All efforts were made to minimize pain and distress. During the study, macaques were observed for general behavior, clinical symptoms, and local reactions at the injection sites twice daily the week after immunizations. When animals were sedated for immunizations or sample collections, body weight and temperature were measured. The age of the animals ranged between 2.5 and 2.8 years, with a mean of 2.6 years and the weight range was between 3.1 to 5.7 kg, with a mean of 3.8 kg. At selected time points, a physical examination was performed by a veterinarian, and clinical chemistry and hematology parameters were measured. Rhesus macaques were negative for tuberculosis and for simian retrovirus (SRV), simian T cell leukemia virus (STLV-1), herpesvirus B, simian immunodeficiency virus (SIV), measles virus and West Nile Virus and Japanese encephalitis virus prior to the study, and have negative fecal culture for *Salmonella*, *Shigella*, *Campylobacter* and *Yersinia*.

Sixteen adult male Chinese *Macaca mulatta* (Rhesus macaque) were selected for this study and animals were pre-screened to discern prior exposure to West Nile or simian immunodeficiency virus. Four rhesus macaque’s were assigned to each of the four vaccine groups as outlined in Table 2 and intramuscular (IM) immunizations were given at weeks 0, 4, 12 and 24. Briefly, Group 1 received DNA vaccine prime (DNA-HIV-PT123, expressing gp140, gag and polnef) in the upper left and right leg and NYVAC-gp140 and NYVAC-Gag-Pol-Nef boost in the left arm. Group 2 received the same DNA prime as Group 1 followed by RepliVax-gp120TM and RepliVax-Gag boost in the right arm. Group 3 received RepliVax-gp120TM and RepliVax-Gag prime in the right arm and NYVAC-gp140 and NYVAC-Gag-Pol-Nef boost in the left arm. Group 4 received NYVAC-gp140 and NYVAC-Gag-Pol-Nef prime in the left arm followed by RepliVax-gp120TM and RepliVax-Gag boost in the right arm. All groups received a total dose of 100 μg protein (50 µg of 1086 and TV1 gp120s each) adjuvanted with MF59 (proteins and adjuvant kindly provided by GSK) and was given in the opposite arm to the viral vector vaccine^38^. At weeks 0, 6, 14, 26 and 36, peripheral blood mononuclear cells (PBMCs) and serum samples were obtained from each immunized animal, and HIV-1-specific T cell and humoral immune responses were analyzed. All assays were performed under Good Clinical Laboratory Practices (GCLP)-compliant conditions.

### Binding antibody multiplex assay (BAMA

The frequency and magnitude of IgG antibody binding were measured by the HIV-1 binding antibody multiplex assay (BAMA) from serum specimens obtained at weeks 0, 6, 14, 26 and 36. HIV-1-specific IgG responses (1:80) against 47 antigens were measured on a Bio-Plex instrument (Bio-Rad) using a standardized custom HIV-1 Luminex assay^21–23,39^. The readout is background-subtracted median fluorescent intensity (MFI), where background refers to an antigen-specific plate level control (i.e., a blank well containing antigen-conjugated beads run on each plate). The positive control was purified polyclonal IgG from HIV-1 infected subjects (HIVIG) using a 10-point standard curve (4PL fit). Serum titrations (1:80, 1:480, 1:2880, 1:17280, 1:103680, 1:622080) were performed to provide a sample within the linear range of the standard curve for calculating area under the titration curve (AUTC). The negative controls were NHS (HIV-1 sero-negative human sera) and blank beads or beads coupled to MulVgp70 His6 (gp70 control). Positive responses against each antigen were determined using MFI in the least diluted samples (1:80). Responses were defined as positive if the following three criteria were met: (i) net MFI (blank-subtracted) value was greater than or equal to an antigen-specific control cutoff; (ii) the MFI value minus the blank value was greater than 3 times the baseline (pre-vaccination) MFI value minus the blank value; and (iii) the MFI value was greater than 3 times the baseline MFI value. AUTC was used to compare response magnitudes. AUTC is the total area of net MFI measurements over the dilution series (1:80, 1:480, 1:2880, 1:17280, 1:103680, 1:622080) calculated using the trapezoid method for each animal by antigen and time point.

### *In vitro* HIV-1 neutralization

Neutralization IC50 titers were measured by HIV-1 neutralizing antibody assay from specimens obtained at weeks 0, 6, 14, 26 and 36. Neutralizing antibodies against HIV-1 were measured as a function of reduction in Tat-regulated luciferase (Luc) reporter gene expression in TZM-bl cells^40–43^. Neutralization titers were measured in pre-and post-immune vaccine sera against panels of HIV-1 clade C tier 1A Env pseudoviruses (MW965.26), HIV-1 clade C tier 2 Env pseudoviruses (TV1.21, 96ZM651.02) and CRF_01 AE Env.IMC.LucR tier 2 virus (C1080.c03). Responses were considered positive if the response titer (ID50) was greater than 20. The ID50 response titer was defined as the sample dilution by which luminescence of virus treated with serum sample is half that of luminescence of untreated virus.

### Antibody-dependent cellular cytotoxicity (ADCC

Sera samples were assessed for the presence of ADCC-mediating antibody responses. Target cells coated with gp120 or infected with HIV-1 Infectious Molecular Clones (Env.IMC.LucR) were tested in a 96-well plate using the GTL and Luciferase ADCC assays, respectively. The gp120-coated or IMC-infected target cells (96ZM651, TV1, or 1086) were incubated along with the study sera samples, the appropriate positive and negative controls (in separate wells) and HuPBMC, and detected through the use of a Granzyme B substrate (GTL)^44^ or Viviren substrate (Luciferase)^45,46^. The percent Granzyme B activity and the percent reduction in RLUs from luciferase activity were measured for each sample at six dilution levels: 100, 400, 1600, 6400, 25600 and 102400. The Granzyme B activity was measured in a single well, while the luciferase activity was measured in duplicates within two separate wells. Positive responses were defined where percent granzyme B activity was greater than 8%. Two ADCC response magnitudes were assessed: (1) the maximum log-transformed dilution (ADCC Ab titer) greater than 8%, and (2) maximum percent granzyme B activity.

### NHP ICS assay

Response rate and the magnitude of HIV-1 specific CD4 and CD8 T cell responses were measured by Intracellular Cytokine Staining (ICS) assay for IFN and IL-2 markers and flow cytometry. Values are reported as the frequency of memory CD4^+^ and CD8^+^ T cells. Cryopreserved PBMCs were stimulated with insert matched clade C HIV-1 epitopes to ZM96gp140 and ZM96gag. This is denoted as Env (T) and Gag (T) peptide pools, which stands for total or combined peptide pools. Cytokine markers (IFN and IL-2) were measured in T cell subsets (total memory CD4^+^ and CD8^+^). As a negative control, DMSO and costimulatory antibodies (anti-CD28 and anti-CD49d) were used as mock samples. In general, samples were excluded from the analysis by the lab, or during processing, if the cell viability is less than 35% or if the total events acquired are less than 35,000. Positive responses were determined using the MIMOSA (Mixture Models for Single-Cell Assays) method^47^. The MIMOSA method uses Bayesian hierarchical mixture models incorporating cell count and cell proportion information to define a positive response by comparing peptides stimulated cells and unstimulated negative controls. MIMOSA estimates the probabilities of peptide-stimulated responses being responders and applies a false-discovery rate multiplicity adjustment procedure^48^ procedure. Responses with false-discovery rate q-values < 0.05 were considered positive. Response magnitudes were compared using background-adjusted percent positive CD4^+^ and CD8^+^ T cells expressing IL2 or INF.

### *In vitro* characterization of innate immunity and T cell immunity of viral vectors

Peripheral blood from healthy adult donors was obtained through the Blood Center of Lausanne, Switzerland. Mononuclear cells were extracted by Ficoll Hypaque (GE Healthcare, Little Chalfont, United Kingdom) density gradient centrifugation. Monocytes (purity > 97%) were isolated by positive selection using magnetic microbeads coupled to an anti-CD14 antibody (Miltenyi Biotech, Bergisch Gladbach, Germany) and cultured in RPMI 1640 medium (Sigma-Aldrich St. Louis, MO) supplemented with 10% fetal calf serum (GE Healthcare), 100 IU/ml penicillin and 100 μg/ml streptomycin (Invitrogen, San Diego, CA). Cells were infected with YF17D, NYVAC-KC, NYVAC-KC-GPN, NYVAC-KC-gp120, RV-Empty, RV-Gag and RV-gp120TM (MOIs 1 and 10). As a positive control, cells were exposed to 10 ng/ml *Salmonella minnesota* ultra-pure lipopolysaccharide (LPS, List Biologicals Laboratories, Campbell, CA). After 18 hours, supernatants were collected and total RNA was extracted using ZR-96 directzol columns (Zymo Research, Irvine, CA). cDNA was synthesized using the QuantiTect reverse transcription kit (Qiagen, Hilden, Germany) and used in real-time PCRs conducted with a QuantStudio™ 12 K Flex system (Life Technologies, Carlsbad, CA). Reactions consisted of 1.25 µl cDNA, 1.25 µl H_2_O, 0.62 µl 10 nM primers and 3.12 µl Fast SYBR® Green Master Mix (Life Technologies). Primer pairs (forward and reverse) were: TCACCAGCACTCGGATGTTC and ACAAGGTATGCGTCTCAGGC (RV), GGGCCAAGCAGGAAAATTGG and ACCTGGCATTCCAGTGTAGC (YF17D), ACAGTGAATCTTGGTTGTAAACTTGAC and TGGTTCGTGGCTCTCTTATCCT (TBP) and GATTTGGTCGTATTGGGCG and CTCGCTCCTGGAAGATGGTG (GAPDH). Samples were tested in triplicate. Calibrators of each target were run in parallel to samples to calculate viral copy numbers per million cells. Supernatants were used to quantify the concentrations of p24 by electrochemiluminescence (ECL Cobas HIV Ag; Roche; Switzerland) and the concentrations of, IFNα, IFNβ, IL-8, and IL-10by Luminex assay (Affimetrix eBioscience, Vienna, Austria).

### Virus-specific CD8 T-cell proliferation

Overnight-rested cryopreserved blood mononuclear cells isolated from one HIV-1-infected elite controller individual (#1010) were stained with 0.25 μM 5,6-carboxyfluorescein succinimidyl ester (CFSE, Molecular Probes, USA) as previously described^49^. Cells were then exposed to RV-Empty, RV-Gag, NYVAC-Empty or NYVAC-GPN vectors at various vector concentrations (0.1, 1 and 10 pfu/cell). In addition, cells were stimulated with GAG peptide (GPNHKARVL; internal control), 200 ng/ml of SEB (positive control; Sigma-Aldrich) or left unstimulated (negative control). At day 6, cells were harvested and stained (4 °C; 20 min) using the aqua LIVE/DEAD stain kit (Invitrogen) and Abs (4 °C; 30 min) to CD3, CD4, and CD8. Cells were fixed with CellFix (BD), acquired on an LSRII SORP (4 lasers: 405, 488, 532 and 633 nm) and frequencies of proliferating CFSE^low^ CD8 T cells were assessed using FlowJo (version 8.8.2; Tree star Inc, Ashland, OR, USA).

### Data analysis and statistics

Statistical analysis of ICS data from immunized mice was done using a previously described methodology to calculate antigen responses over the unstimulated controls^13,50^. Analysis and presentation of distributions were performed using SPICE version 5.1, downloaded from http://exon.niaid.nih.gov ^51^. Comparison of distributions was performed using a Student’s T test and a partial permutation test as previously described^51^. NHP data were analyzed using R statistical software. For each assay, response magnitudes among vaccine responders were compared between experimental groups using Wilcoxon rank-sum test for each time point and antigen or isolate. Tests were only performed if each group had at least 3 responders.

## Supplementary information

Supplementary Information
