# Supplementary material for: Recombinant HIV-1 vaccine candidates based on replication-defective flavivirus vector
Source: Sci Rep. 2019 Dec 27;9:20005. doi: 10.1038/s41598-019-56550-4 (PMC6934588; doi:10.1038/s41598-019-56550-4)

Recombinant HIV-1 vaccine candidates based on replication-defective flavivirus vector

Giel-Moloney M[a*], Esteban M[b] , Oakes BH[a], Vaine M[a], Asbach B[c], Wagner R[c,l], Mize GJ[d], Spies AG[d], McElrath J[d], Perreau M[e], Roger T[f], Ives A[f], Calandra T[f], Weiss D[g], Perdiguero B[b], Kibler K.V[h], Jacobs B[h], Ding S[i], Tomaras GD[j], Montefiori DC[j], Ferrari G[j], Yates NL[j], Roederer M[k], Kao SF[k], Foulds KE[k], Mayer BT[d], Bennett C[d], Gottardo R[d], Parrington M[a], Tartaglia J[a], Phogat S[a], Pantaleo G[e], Kleanthous H[a], Pugachev KV[a]

[a]Sanofi Pasteur, Cambridge, MA 02139, USA;
[b]Centro Nacional de Biotecnología (CNB-CSIC), Madrid, Spain
[c]University of Regensburg (UREG), Institute of Medical Microbiology and Hygiene, 93053 Regensburg, Germany;
[d]Fred Hutchinson Cancer Research Center (FHCRC), Seattle, WA, 98109 USA;
[e]Service of Immunology and Allergy, Department of Medicine, Lausanne University Hospital, 1011 Lausanne, Switzerland;
[f]Infectious Diseases Service, Department of Medicine, Lausanne University Hospital, 1011 Lausanne, Switzerland;
[g]Bioqual Inc
[h]Arizona State University (ASU), Tucson, AZ, 85745 USA;
[i]EuroVacc, Amsterdam, The Netherlands;
[j]Duke University Medical Center, Durham, North Carolina, 27710, USA;
[k]Vaccine Research Center, NIAID, NIH, Bethesda, MD, 20892, USA;
[l]University Hospital Regensburg, Institute of Clinical Microbiology and Hygiene, 93053 Regensburg, Germany

[*]***Corresponding Author***:
Maryann Giel-Moloney, Ph.D.
Sanofi Pasteur
38 Sidney Street
Cambridge, MA 02139
Tel 617-866-4554
email: Maryann.Giel-Moloney@sanofi.com
Office: 617-866-4400
Fax: 617-866-4402

***Keywords**:* HIV-1 vaccine, RepliVax, flavivirus vector, prime-boost, preclinical, nonhuman primate

Table S1. RV and NYVAC vector variants used in the different studies.

**Construct**	**Studies**
RV-Gag (ZM96)	Expression (Fig. S1)<br>Suckling mouse attenuation (Table 1)<br>NHP study (Table 2)<br>*Ex vivo* immunogenicity studies (Fig. 6, Fig. S7)
RV-gp120TM<br>(ZM96)	Expression (Fig. 1)<br>Suckling mouse attenuation (Table 1)<br>Mouse immunogenicity studies (Fig. 2, 3)<br>NHP study (Table 2)<br>*In vitro* immunogenicity studies (Fig. 6, Fig. S7)
RV-Empty	Suckling mouse attenuation (Table 1)<br>Mouse immunogenicity study (Fig. 3)<br>*In vitro* immunogenicity studies (Fig. 6, Fig. S7)
NYVAC-GPN<br>Gag (ZM96)<br>PolNef (CN54)	NHP study (Table 2)<br>*In vitro* immunogenicity studies (Fig. S7)
NYVAC-gp140<br>(ZM96)	Mouse immunogenicity studies (Fig. 2, 3)<br>NHP study (Table 2)
NYVAC-gp145<br>(ZM96)	Mouse immunogenicity study (Fig. 3)
NYVAC-KC-gp140<br>(ZM96)	Suckling mouse attenuation (Table 1)
NYVAC-KC-gp120<br>(ZM96)	*In vitro* immunogenicity study (Fig. 6)
NYVAC-Empty	Mouse immunogenicity study (Fig. 3)
NYVAC-KC-Empty	*In vitro* immunogenicity study (Fig. 6)
NYVAC-KC-GPN<br>Gag (ZM96)<br>PolNef (CN54)	*In vitro* immunogenicity study (Fig. 6)

Supplementary Figure Legends

Fig. S1. *In vitro* replication and expression of RV-Gag construct. (A) Schematic of the RV-Gag genome. **(B)** Stability of the Gag cassette during serial passages to P10 in helper Vero cells (MOI 0.01) as evaluated by determining Gag^+ and WN^+ (total infectious particles) titers at different passages. **(C)** Stability of the Gag cassette as shown by RT-PCR for P10 passage virus using WN vector-specific primers outside of the Gag insert. **(D)** Secreted Gag protein (55 kDa) detected by Western blot in the supernatant of RV-Gag infected Vero (regular) cells as shown for P6 passage virus using rabbit polyclonal anti-Gag antibody (Abcam#63917). Lane 1, molecular weight standard (SeeBlue Plus 2); lanes 2 and 3, RV-Gag unconcentrated and RV-Gag concentrated supernatants, respectively, from RV-Gag-infected cells; lane 4, concentrated supernatant from uninfected cells.

Fig. S2. ADCC responses determined by maximum GzB activity for 1086c delta 7 gp120, TV1 gp120, and 96ZM651_D11 in the four NHP groups. Distributions among positive responses (maximum activity > 8%) are shown as box plots: the mid-line denotes the median, the ends of the box denote the 25th and 75th percentiles, and the whiskers denote the most extreme data points that were no more than 1.5 times the interquartile range. * denotes statistically significant group comparison (Wilcoxon rank-sum, $P < 0.05$). No significant results were observed after multiple test correction.

Fig. S3. Heat map for binding IgG response rates determined against a panel of Env gp120/gp140 and p24 antigens. 100% response rates were observed against all Env gp120/gp140 antigens. Four animals were tested for each group unless otherwise denoted: * = 3 and + = 2.

Fig. S4. Heat map for geometric mean titers (GMT) for area under the titration curve (AUTC) for IgG Env gp120/gp140 binding antibody responses for all four NHP study groups at all study time points. 100% response rates were observed at all time points with peak titers at week 14.

Fig. S5. Heat map for binding IgG response rates by group determined against a panel of V1/V2 and V3 antigens. Four animals were tested for each group unless otherwise denoted: * = 3 and + = 2.

Fig. S6. Recall response as measured by proliferation of virus-specific CD8 T cells from a HIV-1 positive subject. Overnight-rested cryopreserved blood mononuclear cells isolated from one HIV-1-infected elite controller individual were exposed to either RV-Empty, RV-Gag, NYVAC-Empty, or NYVAC-GPN vectors at various concentrations (0.1, 1 and 10 pfu/cell). Controls included Gag peptide, SEB, and unstimulated cells. CD8 T cells were assessed using FlowJo (version 8.8.2; Tree star Inc, Ashland, OR, USA).

Fig. S1. ***In vitro* replication and expression of RV-Gag construct.**

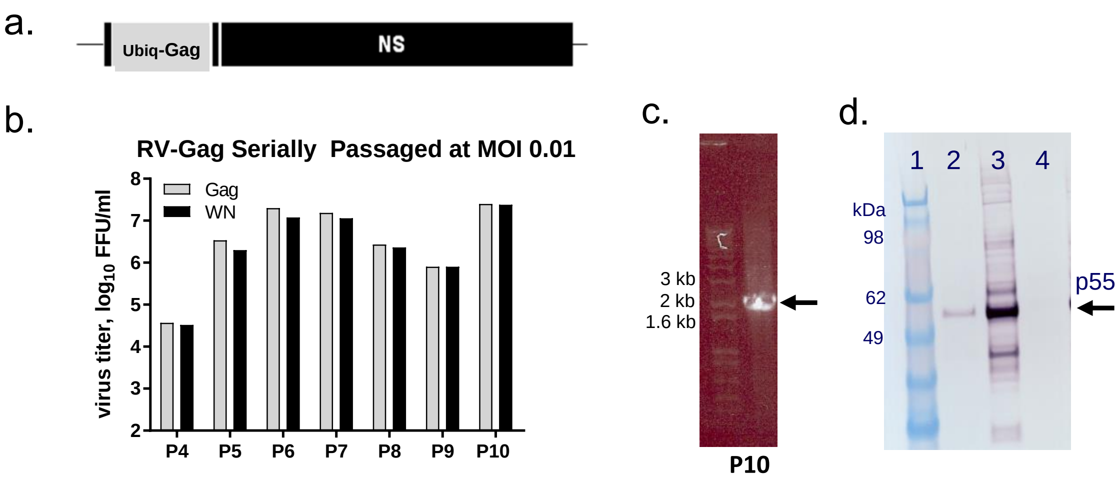

Fig. S2. ADCC responses determined by maximum GzB activity for 1086c delta 7 gp120, TV1 gp120, and 96ZM651_D11 in the four NHP groups.

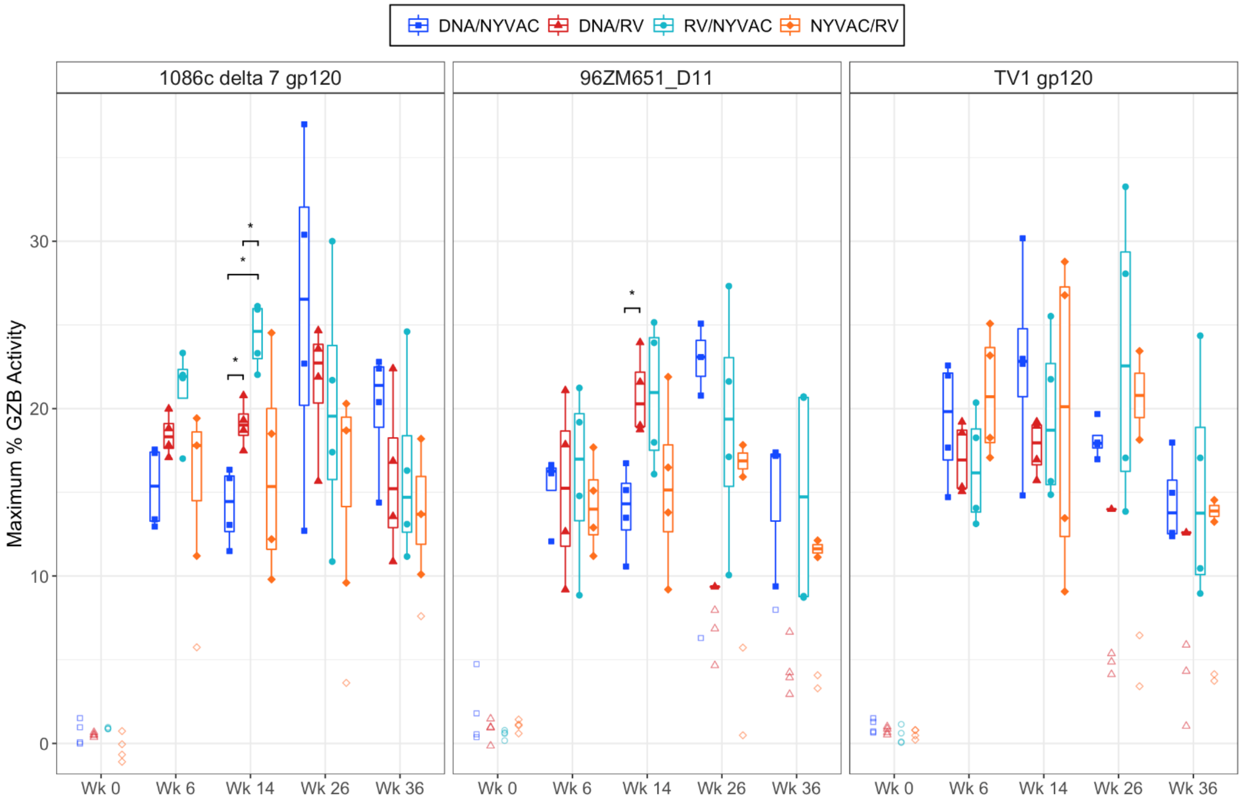

Fig. S3. Heat map for binding IgG response rates determined against a panel of Env gp120/gp140 and p24 antigens.

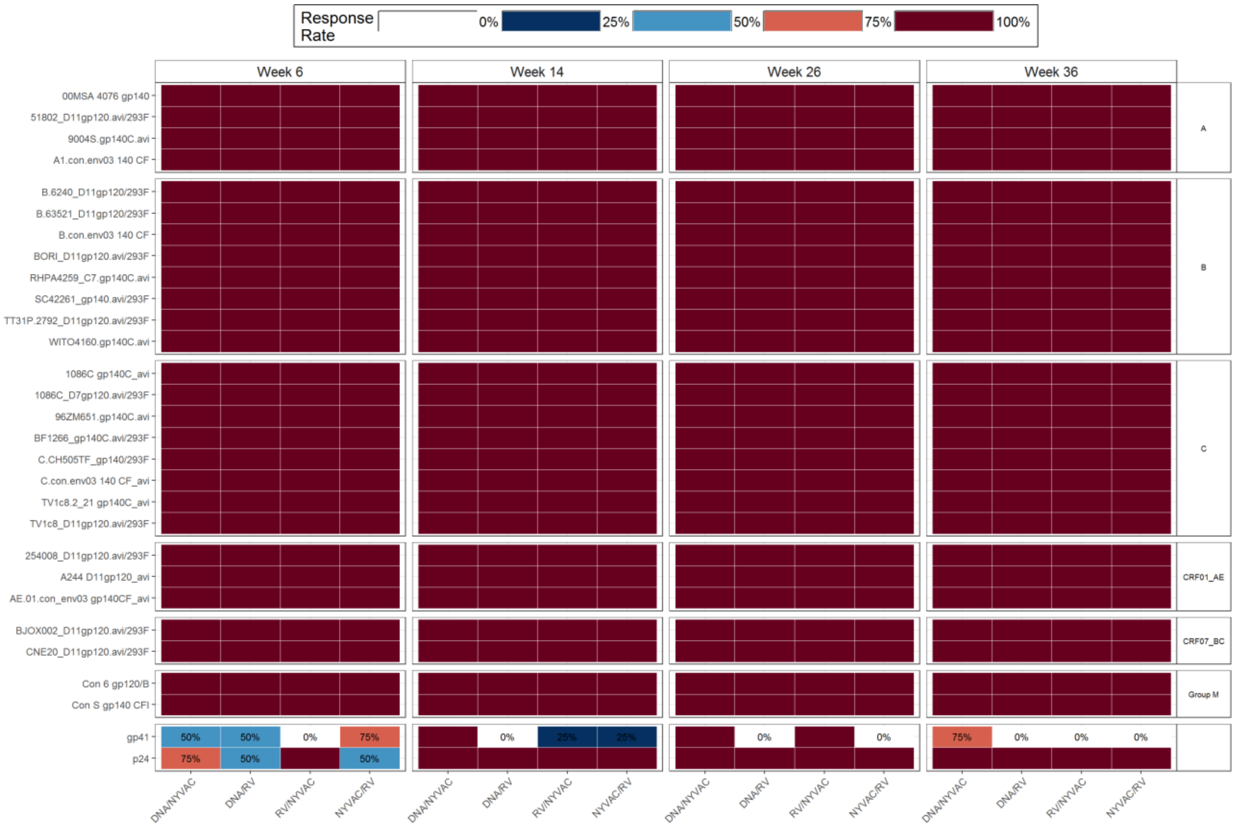

Fig. S4. Heat map for geometric mean titers (GMT) for area under the titration curve (AUTC) for IgG Env gp120/gp140 binding antibody responses for all four NHP study groups at all study time points.

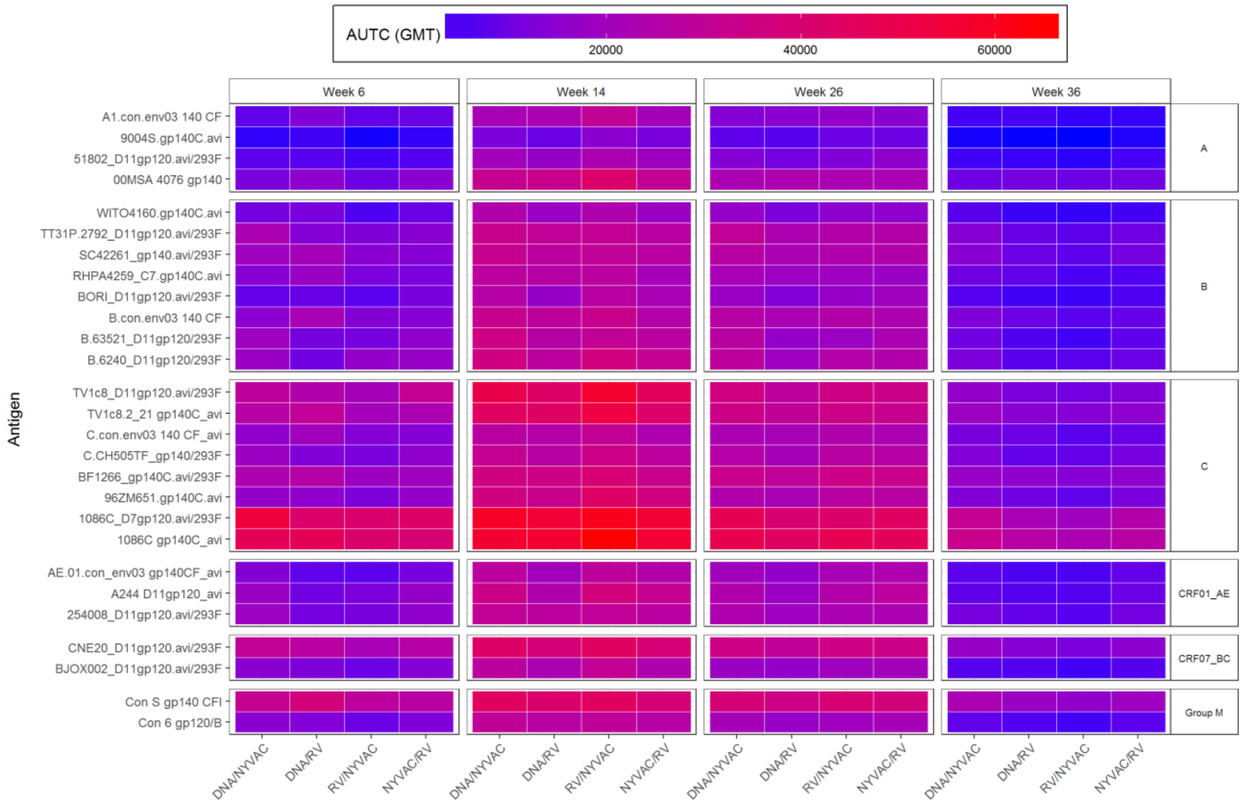

Fig. S5. Heat map for binding IgG response rates by group determined against a panel of V1/V2 and V3 antigens.

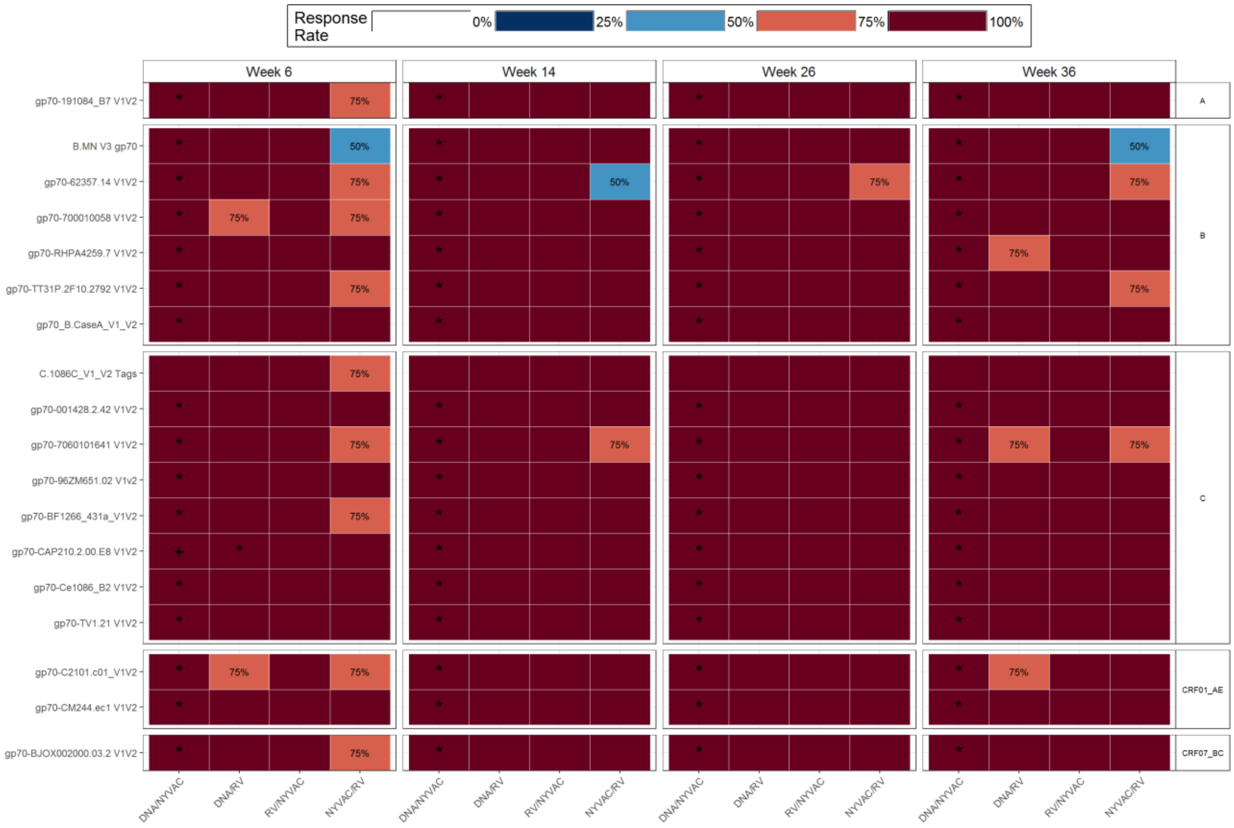

Fig. S6. Recall response as measured by proliferation of virus-specific CD8 T cells from a HIV-1 positive subject.

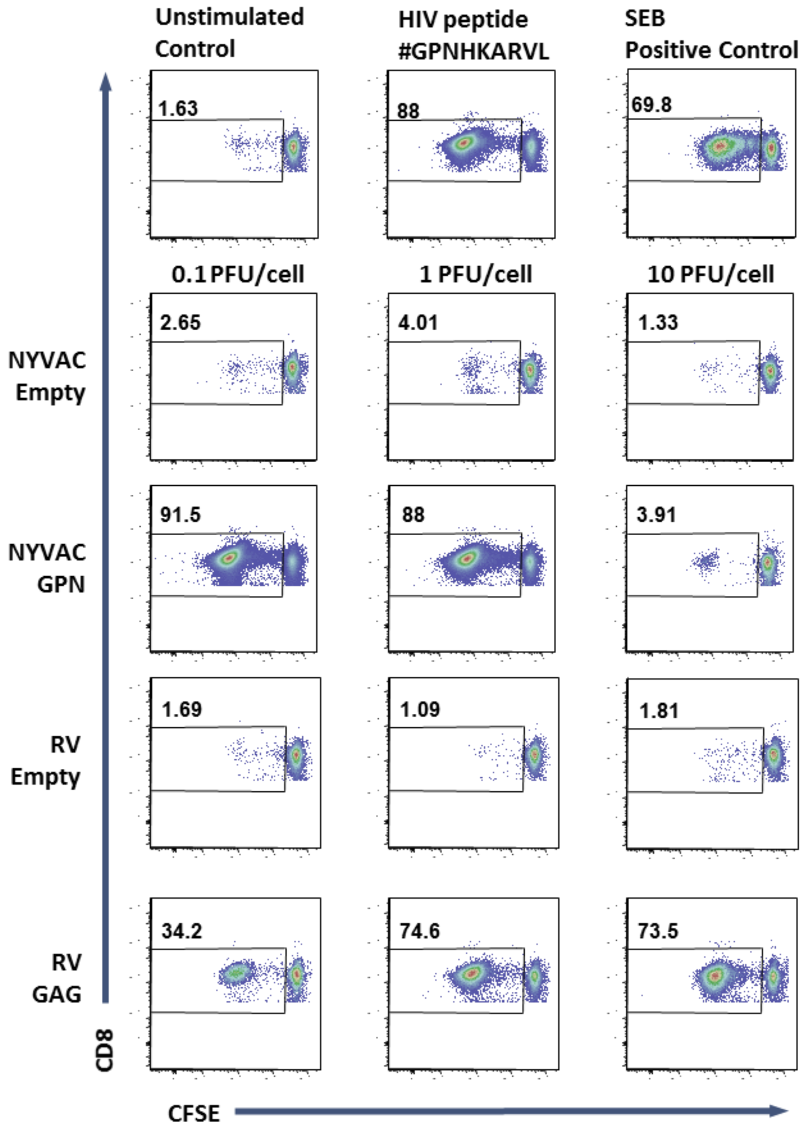

Supplement: Supplementary file 1 — Supplementary Information [file 41598_2019_56550_MOESM1_ESM.pdf]
